# FlorItaly – the portal to the Flora of Italy

**DOI:** 10.3897/phytokeys.156.54023

**Published:** 2020-08-21

**Authors:** Stefano Martellos, Fabrizio Bartolucci, Fabio Conti, Gabriele Galasso, Andrea Moro, Riccardo Pennesi, Lorenzo Peruzzi, Elena Pittao, Pier Luigi Nimis

**Affiliations:** 1 Department of Life Sciences, University of Trieste, Trieste, Italy University of Trieste Trieste Italy; 2 University of Camerino, Camerino, Italy University of Camerino Camerino Italy; 3 Museo di Storia Naturale di Milano, Milan, Italy Museo di Storia Naturale di Milano Milano Italy; 4 University of Pisa, Pisa, Italy University of Pisa Pisa Italy

**Keywords:** biodiversity data, checklist, floristics, taxonomic standard

## Abstract

Digital data concerning the flora of Italy are largely fragmented among different resources hosted on different platforms, and often with different data standards, which are neither connected by a common access point, nor by web services, thus constituting a relevant obstacle to data access and usage. Taxonomic incongruences add a further complication. This paper describes “FlorItaly”, an online information system which allows to access and query updated information on the checklist of the flora of Italy, aiming at becoming an aggregator for Italian botanical resources. “FlorItaly” was developed in a collaborative effort by more than 50 taxonomists, with the support of the Italian Botanical Society, and of Project “Dryades” (University of Trieste), to provide a better and reliable organization of botanical knowledge in Italy, as well as a relevant simplification for data retrieval, and a further stimulus towards a more collaborative approach in botanical research.

## Introduction

Taxonomic checklists summarize the hitherto available knowledge of the biodiversity in a given area. They can be simple lists of names, or provide detailed information on each taxon. In well-explored areas, they are the basis for the development of a true flora, while in poorly known areas they provide a baseline for further investigation. Furthermore, checklists are also a mean for nomenclatural stability, providing a reliable taxonomic backbone. Checklists can potentially be of use for connecting information from different sources, ideally making biodiversity data interoperable through names (or Unique Identifiers – UIDs – associated with names). While being a fundamental tool for researchers, they can be relevant also for decision-makers, as they provide a baseline for informed decisions. National checklists are especially relevant, since environmental decisions are taken at national level. Being open-ended works, checklists can greatly benefit from a digital “publication”, which allows their updating with new information, continuously, or at regular intervals. Digital, online publication also makes a checklist more visible and accessible to target users (Martellos 2012). Plus, providing open access to information has several positive effects, such as facilitating research, avoiding duplication of efforts, stimulating contributions, etc. (Berendsohn et al. 2018)

The development of online plant data repositories, especially focused on taxonomic information as nomenclatural backbones, is the main aim of several international initiatives. In Europe, the Euro+Med PlantBase (Euro+Med 2006) is a long-lasting effort which aims at achieving an agreed taxonomic core for all families, genera, species, subspecies and, where appropriate, cultivars described from the Euro-Mediterranean region, involving experts from all over Europe. The Plants of the World Online portal (POWO 2020) has a broader aim, since it focuses on enabling users to access information on all seed-bearing plants known worldwide by 2020. It is part of the Science Strategy of Royal Botanic Gardens, Kew, which aims at disseminating Kew’s scientific knowledge of plants and fungi to maximize its impact in science, education, conservation policy and management. Another “global” effort is the World Flora Online (WFO 2020), a project carried on since 2010 in the framework of the Global Strategy for Plant Conservation (GSPC) of the U.N., and supported by the Conference of the Parties to the Convention on Biological Diversity. It aims at becoming a Web-based compendium of the world’s plant species, as a first step towards the development of a consolidated global information service on the world’s flora.

In Italy, efforts for the creation of a network of databases on the flora and vegetation of the country date back from far before the Rio Conference. As discussed by Nimis et al. (1984), Italy was one of the first countries in which a relevant, cooperative effort aimed at creating a distributed network of digital resources for botanical data was initiated. A taxonomic backbone, the first edition of the Flora of Italy (Pignatti 1982) was databased, to serve as a central aggregation core for other databases, especially vegetation data. The authors of that effort already foresaw future steps, such as the integration of digital identification keys in the network. Their vision, however, had not the luck it deserved, and presently Italy still lacks a national network of databases for botanical data. In 2005, the first edition of the checklist of the Italian vascular flora (Conti et al. 2005) was an opportunity for members of the Working Group “Floristics, Systematics, and Evolution” of the Italian Botanical Society to establish an effective network of scientific collaborations (Peruzzi 2018) for updating the list and distributional data up to 2007 (Conti et al. 2007). Other important collaborative efforts followed (Celesti-Grapow et al. 2009a, b, 2010; Peruzzi et al. 2015, 2019; Brundu et al. 2017; Orsenigo et al. 2018), so that – after 13 years – the time was ripe for an updating of taxonomic, and geographic knowledge on the Italian vascular flora. Thanks to the close cooperation of more than 50 authors, two separate new checklists were published in 2018: one of the native (and doubtfully native) vascular flora (Bartolucci et al. 2018a), the other concerning alien taxa (archaeo- and neophytes) (Galasso et al. 2018a). Thanks to Project “Dryades” of the University of Trieste (Nimis et al. 2003; Nimis and Martellos 2009; Martellos and Nimis 2015), a few months after their publication, nomenclatural, taxonomical and distributional data, integrated with their first updates (Bartolucci et al. 2018b, c, 2019a, b; Galasso et al. 2018b, c, 2019a, b), were organized into an information system on the vascular flora of Italy.

This paper details the result of this effort, “FlorItaly”, which is accessible online at the address http://dryades.units.it/floritaly, and is being updated every six months.

## Materials and methods

The core of “FlorItaly” is a software written in PHP language, which works on data stored in a MySQL database, running on the servers of the Project “Dryades”, hosted at the Department of Life Sciences, University of Trieste. “FlorItaly” organizes nomenclatural and distribution data from the recent checklists of the Italian native and alien vascular flora and their subsequent updates, which are published every six months, and makes them interoperable with other resources.

### Checklist data

The taxonomic circumscription of families follows PPG I (2016) for ferns and fern allies, Christenhusz et al. (2011b) for Gymnosperms, and Angiosperm Phylogeny Group (2016) for Angiosperms, with the exception of Dipsacales (Reveal 2011), Caryophyllales (Hernández-Ledesma et al. 2015) and Boraginales (Luebert et al. 2016). Authors’ citations of plant names were standardized following the Rec. 46A Note 1 of the ICN (McNeill et al. 2012), i.e. according to IPNI (2012). The checklist includes also apomictic taxa belonging to *Alchemilla* and *Rubus* (Rosaceae), *Hieracium*, *Pilosella* and *Taraxacum* (Asteraceae), and the *Ranunculus auricomus*-complex (Ranunculaceae). Taxa at varietal rank and hybrids were not considered. The system organizes 10,898 infra-generic taxa, and 12,887 synonyms, plus 64,001 vernacular names, and an archive of more than 220,000 digital images.

The main data source of “FlorItaly” are the two checklists of the native (Bartolucci et al. 2018a), and alien (Galasso et al. 2018a) vascular flora of Italy, which are updated every six months by a team of researchers of the Working Group “Floristics, Systematics, and Evolution” of the Italian Botanical Society (Bartolucci et al. 2018b, c, 2019a, b; Galasso et al. 2018b, c, 2019a, b). The updates, regularly published on the journal “Italian Botanist”, are integrated in the online version immediately after their publication. Each new online version is labeled with the year plus an “a” or “b”, depending on the semester of the update.

Distribution data (taken from the checklists, and expressed as presence-absence) are given for each of the 20 administrative regions of Italy (two enclave-countries Republic of San Marino and Vatican City State are not considered). When information on the occurrence of a given subspecies for a region is missing, only the occurrence at species level is reported. For each region, the presence and occurrence status of each taxon is provided by using the following categories: a) occurring, b) doubtfully occurring, c) no longer recorded (reliable historical record), d) extinct or possibly extinct, e) recorded by mistake, f) alien at regional and/or national level (casual, naturalized, invasive, undefined invasion status), g) Italian endemic (status attributed to those taxa occurring only in Italy, or in Italy and Corsica, or in Italy and Malta), h) cryptogenic, i.e. a doubtfully native taxon, whose origin in Italy is unknown, i) taxonomically doubtful, j) data deficient (unknown regional distribution; unknown alien status), k) archaeophyte, and l) neophyte. Occurrence status can also be provided at national level, when relevant, with the following categories: i) confirmed/not confirmed; ii) extinct; iii) doubtful; iv) data deficient; v) erroneously reported for the country); vi) endemic; vii) cryptogenic; viii) esoticity (neo- or archaeophyte). Presence and occurrence status at regional level are depicted in a distribution map (see description of a taxon page below), while those at national level are reported as textual information. A national standardized system was developed by Celesti-Grapow et al. (2009a, b, 2010) to identify taxa alien to Italy. Definitions used in the system were provided by Pyšek et al. (2004):

• **casual**: alien plants that may thrive and even produce offspring occasionally outside cultivation, but that usually disappear, since they are unable to form self-maintaining populations. Hence their persistence relies on repeated introductions;

• **naturalized**: alien plants that occur with self-maintaining populations without direct human intervention;

• **invasive**: alien plants that occur with self-maintaining populations without direct human intervention, and produce fertile offspring which can reach considerable distances from the parent individuals, thus being able to spread over a large area;

• **archaeophytes**: alien plants introduced to Italy before 1492 (approximate date corresponding to the discovery of America);

• **neophytes**: alien plants introduced to Italy after 1492.

Taxa involved in former domestication processes are separated into two categories:

• **culton**: plant distinct from its wild relative(s) and capable to conserve its taxonomic independence in cultivation only; records from the wild are regarded as casual occurrences;

• **feral**: wild plant originated from a culton escaped from domestication, and usually taxonomically distinct from its wild relative; it can either belong to the same taxon of the culton or to a different taxon.

Taxa at varietal rank were not considered; hybrids were considered only for the alien flora.

All of these data are released under a under a CC BY-SA 4.0 license.

### Interoperability with other resources

“FlorItaly” makes the checklists data inter-operable with other resources (see below for a comprehensive list) which use the same taxonomy and the same UIDs of the checklist. This is achieved through:

• web services. “FlorItaly” uses a web service embedded into Wikiplantbase (Bagella et al. 2015; Peruzzi and Bedini 2015; Barberis et al. 2016; Domina et al. 2016) to retrieve information on whether it hosts data for a given infrageneric taxon, and generates a link to the Wikiplantbase taxon page, reporting the number of available records. Queries are sent out encoded in KVP format, and results are retrieved encoded in json format. Web services are also used to access some of the “Dryades” resources, especially image archives. In this case, queries are sent out encoded in KVP format, and results are retrieved in XML format.

• direct querying to external databases, in the case of several “Dryades” resources. Since the developers of all the resources of “Dryades” and “FlorItaly” are the same, it was possible to directly access the other databases by querying through taxon name.

• auto-generated links, made to provide access to resources of Acta Plantarum (2020). A link is dynamically generated by using the UID of each taxon in the checklist. This provides access to the I.P.F.I. (Index of Plants of the Flora of Italy) taxon page.

## Results

“FlorItaly” is accessible online since June 20^th^, 2018, at the address: http://dryades.units.it/floritaly. It has an average of 2,200 page views, and 220 unique visitors per day, and a total of ca. 1,100,000 page loads since its publication on the Internet. Users’ retention rate – calculated for March 2020 – is 61%.

### The information system

“FlorItaly” has 3 query interfaces: 1) basic, 2) standard, and 3) advanced. In the second and third interface, users are allowed to combine several parameters in order to perform complex queries. The combination of parameters is transparent to the users, and makes use of the two logical operators OR (when two or more parameters are selected, if either is true, the complex expression is true) and AND (when two or more parameters are selected, all of them must be true for the complex expression to be true). The two operators are combined in the queries differently in each query interface (see below). All interfaces allow to query by taxon name. The query can be done by inputting an accepted name, a synonym, or part of their names. The query is case-insensitive, and no special characters are allowed (e.g., querying from a string followed by the character “*” will return no results, since the character “*” is read as text, and not as a “jolly” character). Each interface always returns a list of accepted names and/or synonyms, each giving access to a taxon page.

***Basic query interface***. It allows to quickly access all the information on each taxon, organized in “taxon pages”, which are dynamically generated from the database. When a synonym is typed as a query string, the thesaurus of synonyms is invoked, providing a link to the accepted name. For each query, a list of all synonyms, if present, is provided after the list of the accepted names. The basic interface also allows to filter the query by family (selecting one family from a drop-down menu). Furthermore, it permits to query the Thesaurus of Italian and local names of Project “Dryades”. When an Italian name is used as query string, the system provides a list of vernacular names together with the related scientific name(s). The latter give access to the taxon pages.

***Standard query interface***. This interface allows simple queries on national and regional floras by including/excluding: i) taxa known from reliable historical records only; ii) extinct taxa; iii) taxa reported by mistake; iv) taxa known from doubtful records only; v) alien taxa. The first four parameters are combined by the logical operator OR (in the same query users can include/exclude more than one of them). The last parameter (alien taxa) is combined with the others by logical operator AND. This interface can also display the results in the form of an image gallery.

***Advanced query interface***. This interface allows complex queries on the flora of the whole country, or on the floras of different Operational Geographical Units (OGUs), consisting of one or more administrative regions. If no OGU is selected, the system operates on the whole national flora, including extinct taxa, and those reported by mistake. For Italy, or for any other OGU, it is possible to refine the query by using the following seven groups of parameters:

A) occurrence status: 1) taxa known from reliable historical records only; 2) extinct taxa; 3) taxa reported by mistake; 4) taxa known from doubtful records only; 5) data deficient taxa (those recorded from Italy, but without sufficient knowledge on regional records);

B) alien status: 6) invasive; 7) naturalized; 8) casual; 9) other alien taxa (currently without invasiveness status); 10) cryptogenic taxa (doubtfully native);

C) alien, by period of introduction: 11) neophytes (since 1492); 12) archaeophytes (until 1492);

D) feral/culton status: 13) feral; 14) culton;

E) 15) Italian endemics;

F) 16) exclusive endemics (Italian endemics whose distribution is restricted to the selected OGU);

G) 17) taxonomically doubtful taxa.

Inside each group of parameters, the systems uses the logical operator OR, while among the groups the system uses the logical operator AND, as described for the standard query interface (see above).

### Taxon pages and external resources

**Figure 1.**
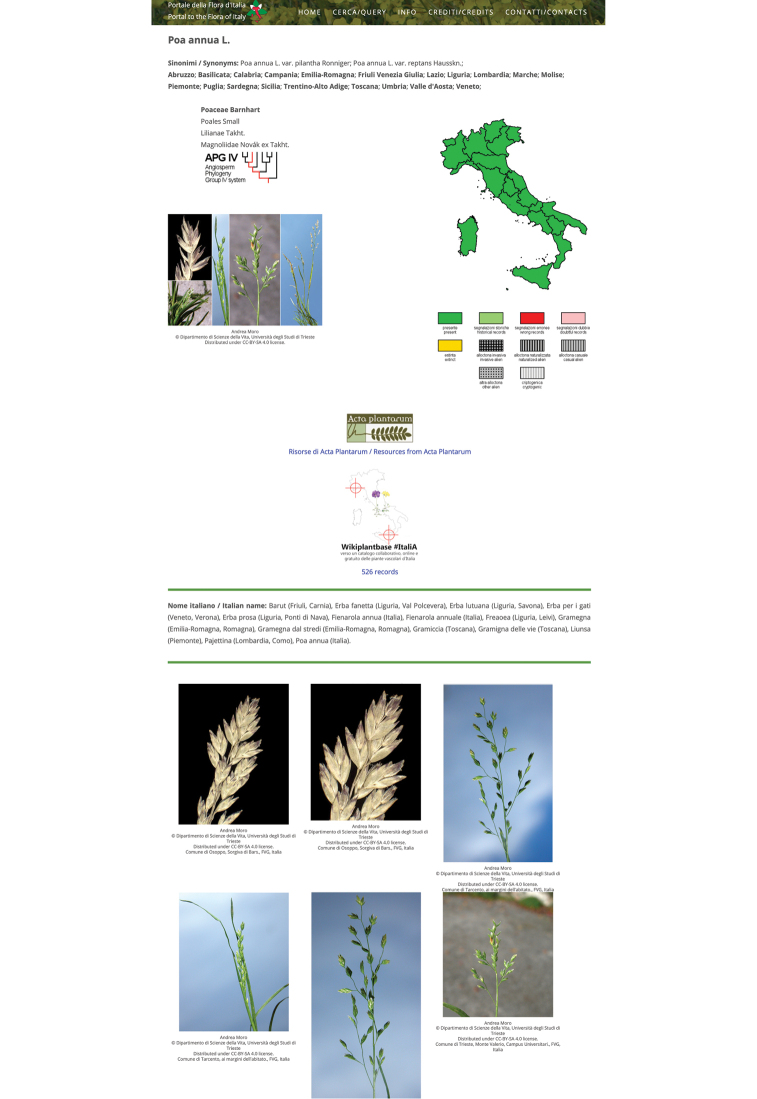
Taxon page for *Poa annua* L. from *FlorItaly* [accessed on 18 June 2020]. The page lists taxon name, synonyms, distribution, also depicted in a distributional map, taxonomic position (in this case in the APG IV scheme), an image, links to external resources, Italian names, and a photo gallery.

The taxon pages, which are the final outcome of a query, display data from the checklist, and aggregate, or link different external resources (Fig. 1).

At the top of the taxon page there is the accepted name, followed by synonyms, and the presence in the 20 administrative subdivisions of the country. Other information, such as whether the taxon is an archaeo-/neophyte, endemic, etc. are reported immediately below, when present.

This first block is followed by two external resources, a link to a cladogram, with the relative taxonomic information, and an image, which are displayed on the left. On the right of the page, a distribution map is dynamically generated by using the presence in the administrative regions, plus the other data on the taxon (e.g. whether it is an invasive).

The cladograms and the taxonomic information are external resources, deriving from an archive of cladograms from Project “Dryades”, which follows Smith et al. (2006), Schuettpelz and Pryer (2008), Ran et al. (2010), Christenhusz et al. (2011a, 2011b), and the Angiosperm Phylogeny Group (2016). The image comes from the archive of digital images of vascular plants of Project “Dryades” (see: http://dryades.units.it/cercapiante/index.php).

These two sections are followed by a link to the I.P.F.I. database of Acta Plantarum, and a link to the Wikiplantbase Italy project. Each link reports the name of the resource, and its logo, and opens in a new page. Acta Plantarum is one of the most active groups of amateur botanists in Italy, and the resources present in the I.P.F.I. pages include digital images, altitudinal distribution, growth forms, etymology, discussions, etc. The Wikiplantbase initiatives (Bagella et al. 2015; Peruzzi and Bedini 2015; Barberis et al. 2016; Domina et al. 2016) collect and organize geo-referenced occurrence data (Bedini et al. 2016; Peruzzi et al. 2017) for different administrative regions of Italy. Originally, “FlorItaly” linked each of the regional initiatives, but, since they are growing in number, it was decided to switch to a link to the national Wikiplantbase portal, which aggregates data from all regional initiatives. These links are followed by names from the Thesaurus of vernacular names for Italy of Project “Dryades”, largely based on those proposed by Pignatti (1982), and especially by Penzig (1924).

At the bottom of the taxon page there is an image gallery, which currently derives from the archives of Project “Dryades”. However, since “FlorItaly” can be made interoperable with other resources, potentially other archives can be accessed, and integrated in the taxon pages (or linked as external resources). Images are displayed as thumbnails, and can be enlarged by a simple click. Each image is coupled with metadata that specify author, source, license of use, locality, and date of the shot, and any other metadata, when available. When the license is not specified, the author of the image reserves all rights. Approximately 80% of the images of Project “Dryades” are original, and released under a CC BY-SA 4.0 license, which means that anyone can use them, for any purpose, provided that author, source and license are mentioned.

## Discussion

Global access to biodiversity information is considered mandatory for research, and decision making since the UNESCO Conference of Rio outputs (Berendsohn and Seltmann 2010; Berendsohn et al. 2010; Berents et al. 2010; Vollmar et al. 2010). Furthermore, the Convention on Biological Diversity includes as its Target 1 the need for “An online flora of all known plants”. Several important actions have been taken in the last 20 years to digitize and mobilize biodiversity data. Large distributed databases (e.g., GBIF, http://www.gbif.org, or BioCASE, Güntsch et al. 2007) have been created to organize and share primary biodiversity data, and several approaches to digital identification have been investigated (Dallwitz et al. 2002; Martellos 2010; Nimis and Vignes Lebbe 2010). The digitization of checklists has been also addressed. One of the first efforts to publish online national checklists was the “Index Synonymique de la Flore de France” (Brisse and Kerguélen 1994; Kerguélen 1994; https://www2.dijon.inra.fr/flore-france/). More recently, thanks to the EDIT platform for cybertaxonomy (Berendsohn 2010), a number of checklists were published online (https://cybertaxonomy.eu/references): the second edition of the Flora of Greece, the Flore du Gabon, the Flora of Cyprus, the Flora of Central Africa, etc.

Given the relevance of the two checklists of native and alien vascular flora of Italy (Bartolucci et al. 2018a; Galasso et al. 2018a), the mobilization of their rich content after their publication in paper-printed form was a further step (Peruzzi 2018). Data mobilization is the focus of several efforts in biodiversity informatics, since it became evident that data stored in the form of journal articles need to be extracted, organized in line with appropriate standards, and aggregated into online databases. Some recent examples in this direction are the BIOfid information service (Driller et al. 2018), the PLAZI workflow (Agosti et al. 2019), and the Open Biodiversity Knowledge Management System (OBKMS) initiative (Penev et al. 2018). For the creation of “FlorItaly”, a collaborative effort was initiated between Project “Dryades” of the University of Trieste (Nimis et al. 2003), and the leading authors of the two checklists, in order to make their data accessible online as an actual information system capable of complex queries, and as a “core” for aggregating, and linking further data and resources. In Italy, botanical digital data are currently fragmented in a wealth of different resources, and the role of “FlorItaly” as an aggregator could become very relevant in the next years. As an example, primary biodiversity data for plants are available in the Wikiplantbase repositories, in the Italian Biodiversity Network of the Ministry of Environment (Martellos et al. 2011), and in several online herbaria (e.g., the Virtual Herbarium of the University of Palermo, http://www.ortobotanico.unipa.it/virtual_herbarium.html), often hosted on different platforms, and adopting different standards, thus making access to data quite complex and fragmented, and creating a relevant obstacle to data retrieval. Taxonomic incongruences add a further complication. The use of a checklist as an aggregator provides a necessary taxonomic backbone to all other resources. All members of the Working Group are committed on agreeing on a common taxonomic backbone (see data and methods), adopted at the national level. The use of “FlorItaly” as an aggregator provides a better and reliable organization of botanical knowledge in the country, as well as a relevant simplification for accessing data by researchers. At the same time, it will provide a further stimulus towards a more collaborative approach in botanical research, allowing quick and solid answers to challenging questions, especially now that global change-related issues require fast and reliable answers from science.

As far as sustainability of the system is concerned, “FlorItaly” will be maintained by the Department of Life Sciences of the University of Trieste, which will assure regular updates of software, and data. Furthermore, a backup instance of “FlorItaly” will be installed in the forthcoming LifeWatch (Basset and Los 2012) Center for Botanic Diversity Data, which will be hosted at the Department of Biological, Geological, and Environmental Sciences, “Alma Mater Studiorum” University, Bologna. As far as data are concerned, sustainability and regular updates are provided by the volunteer, collaborative work of the members of the Working Group “Floristics, Systematics, and Evolution” of the Italian Botanical Society, which come from the academia, or are private citizens, committed in a medium-long term effort.

While originally developed to target an academic audience, “FlorItaly” can be useful for a wider target audience, such as decision makers, and citizens, in the fields of formal education, life-long learning, and citizen science. Other resources (such as primary biodiversity repositories, a *loci classici* database, etc.) are planned to be made interoperable in “FlorItaly”. Furthermore, the system could be potentially connected to digital identification keys. Some keys have been already developed in the framework of Project “Dryades”, by using software FRIDA (Martellos 2010). These keys are particularly suitable to be connected to “FlorItaly”, since FRIDA allows to generate keys to lists of taxa (local floras, or any group of plants, e.g., aquatic plants of N Italy), such as those which result from querying “FlorItaly”. Thus, potentially, any query could produce not only a mere list of taxa, but a digital identification key to those taxa as well. Other planned changes in the system will be introduced in the next technical releases. They will be decided on the basis of users’ feedback, and will focus on improved usability of the interfaces, and on the possibility of changing query parameters in the results page, without the need of performing another query. Furthermore, the possibility to download results data in csv format will be also explored.
